# MiR-138 Suppresses Cell Proliferation by Targeting Bag-1 in Gallbladder Carcinoma

**DOI:** 10.1371/journal.pone.0126499

**Published:** 2015-05-11

**Authors:** Fei Ma, Mingdi Zhang, Wei Gong, Mingzhe Weng, Zhiwei Quan

**Affiliations:** 1 Department of Oncology, Xinhua Hospital Affiliated to Shanghai Jiaotong University School of Medicine, Shanghai, China; 2 Department of Surgery, Xinhua Hospital Affiliated to Shanghai Jiaotong University School of Medicine, Shanghai, China; Cedars-Sinai Medical Center, UNITED STATES

## Abstract

**Background:**

MiR-138 is frequently downregulated in different cancer types and is thought to be involved in the progression of tumorigenesis. However, the molecular mechanism of miR-138 involvement in gallbladder carcinoma still remains unknown.

**Methods:**

The expression of miR-138 in 49 gallbladder carcinoma samples and paired normal gallbladder samples was analyzed using quantitative reverse transcription–polymerase chain reaction. The biological functions of miR-138 and Bag-1 (Bcl-2-associated athanogene-1) on cell proliferation were examined using 3-(4, 5-dimethylthiazolyl-2)-2,5-diphenyltetrazolium bromide and apoptosis assays. Targets of miR-138 were predicted using bioinformatics and validated using luciferase reporter and Western blot analyses. The in vivo effects of miR-138 were examined using subcutaneous inoculation of gallbladder carcinoma cells in Balb/c nude mice.

**Results:**

Compared with their paired normal gallbladder samples, the gallbladder carcinoma samples had decreased expression of miR-138 and increased expression of Bag-1. Overexpression of miR-138 inhibited the proliferation of gallbladder carcinoma cells. Bag-1 was defined as a novel target of miR-138. Both the inhibition of Bag-1 by miR-138 and the silencing of Bag-1 by siRNA led to alterations of apoptosis-related proteins such as Bcl-2 and Bax. Restoring expression of Bag-1 eliminates the effects of miR-138 on cell proliferation and apoptosis. Furthermore, overexpression of miR-138 markedly inhibited the growth of tumors in the gallbladder carcinoma xenograft model in nude mice.

**Conclusions:**

Expression of miR-138 is frequently reduced in gallbladder carcinoma when compared to normal cells. Overexpression of miR-138 inhibited cell proliferation by directly suppressing the expression of Bag-1. These results suggest that miR-138 plays an important role in inhibiting the growth of gallbladder carcinoma.

## Introduction

Gallbladder carcinoma is the most common malignancy of the bile duct, and it is very aggressive, resulting in dismal prognosis and high death rates [1]. Although advancements have been made in the treatment (surgery, radiotherapy, and chemotherapy) of gallbladder carcinoma in recent decades, the 5-year survival rate of patients with gallbladder carcinoma remains low [2–5]. During tumor progression, many genetic and epigenetic changes occur, leading to uncontrolled malignant growth and cell division [6]. Therefore, improved insight into the molecular mechanisms of gallbladder carcinoma proliferation may offer a more effective treatment; thus, improving prognosis.

MicroRNAs (miRNAs) are small, single-stranded, endogenous, and noncoding RNAs that are capable of regulating the expression of genes at both the transcriptional and translational levels [7, 8]. MiRNAs with perfect or near-perfect complementarity to the cognate sequence 3′-untranslated regions (UTRs) of specific mRNAs repress translation from mRNA to protein or induce mRNA cleavage, thereby, regulating the expression of target genes [7, 9]. Research studies have showed that miRNAs are involved in a wide variety of biological processes including cell proliferation, apoptosis, differentiation, and tumor initiation and promotion. Thus, identifying these miRNAs may provide new insights into the genesis and progression of cancer [10–13]. Since miRNA recognizes the short fragment in the 3′-UTR of mRNA with imperfect complementarity, a miRNA can act as an oncogene or a tumor suppressor gene in different types of cancer through different targeted genes [9, 14–19]. However, there is limited information regarding the potential role of miRNA dysregulation in gallbladder carcinoma.

MiR-138 plays an important role in different types of cancer and functions as a tumor suppressor gene. It is downregulated in nasopharyngeal carcinoma specimens and nasopharyngeal carcinoma cell lines. The overexpression of miR-138 inhibits cell proliferation and colony formation [20]. Downregulation of miR-138 in neuroblastoma and thyroid carcinoma is associated with the human telomerase reverse transcriptase (hTERT), which promotes malignant cell growth of many tumors [21, 22]. Recent studies have indicated that miR-138 is frequently reduced in leukemia and lung cancer and associated with drug resistance [23, 24]. However, to our knowledge, its expression and biological roles in gallbladder carcinoma remain unclear.

In this study, we found that the expression of miR-138 was significantly lower in gallbladder carcinoma specimens. Furthermore, overexpression of miR-138 inhibits cell growth *in vitro* and the growth of tumors *in vivo* and is associated with cell cycle arrest. It was also identified that Bag-1 (Bcl-2-associated athanogene-1) is a direct and functional target of miR-138 in gallbladder carcinoma.

## Materials and Methods

### Patient tissue samples

A total of 49 surgical specimens of cancerous tissues and their paired adjacent non-neoplastic tissues were obtained from patients with gallbladder carcinoma who underwent surgery between 2007 and 2009 at Xinhua Hospital affiliated to Medical School of Shanghai Jiaotong University. Two pathologists independently assessed the histo-pathological diagnosis and differentiation based on the World Health Organization classification system. Fresh specimens were immediately frozen in liquid nitrogen after resection. All patients provided written informed consent for the use of their tumor tissues for clinical research, and the project protocols were approved by the Medical Ethics Committee of Xinhua Hospital Affiliated to Shanghai Jiaotong University School Of Medicine with the permit number of 120614SHJT06.

### Cell lines and transfection

The human gallbladder carcinoma cell lines OCUG-1 and NOZ were cultured in RPMI-1640 (Gibco, China). A human embryonic kidney cell line (HEK293T), purchased from the Shanghai Cell Bank (Shanghai, China), was grown in Dulbecco’s modified Eagle’s medium (Hyclone, China). All media were supplemented with 10% fetal bovine serum (Gibco, Carlsbad, CA), 100 IU/mL penicillin and streptomycin (Sigma, St. Louis, MO), and incubated at 37°C in a humidified incubator with an atmosphere of 5% CO_2_.

In order to silence Bag-1, Bag-1 small interfering RNA (siRNA) and its negative control oligonucleotide were purchased from Genechem (Shanghai, China). The siRNA targeted the following sequences: 5′-AATTCCG CTCCAGAGACGGTA-3′ (Bag-1) and 5′-CGTGATCTTCACCGACAAGAT-3′ (control). Transfections were performed using Lipofectamine2000 (Invitrogen, Carlsbad, CA) according to the manufacturer’s instructions. The transfected cells were resuspended and cultured in regular culture medium for 48 to 72 hours before analysis.

### Construction of vectors

The oligonucleotides encoding the pre-miR-138 sequence were synthesized as follows: 5′-AATTCCCCTGGCATGGTGTGGTGGGGCAGCTGGTGTTGTGAATCAGGCCGTTGCCAATCAGAGAACGGCTACTTCACAACACCAGGGCCACACCACACTACAGGG-3′ and 5′-CCTGTAGTGTGGTGTGGCCCTGGTGTTGTGAAGTAGCCG TTCTCTGATTGGCAACGGCCTGATTCACAACACCAGCTGCCCCACCACACCATGCCAGGGG-3′. The oligonucleotides were annealed and then, cloned into pCDH-CMV-MCS-EF1-copGFP vector (SBI, Mountain View, CA, USA) between the EcoRI and BamHI sites. LentiVirus packaging and infection were performed according to the recommendations of the manufacturer.

The full-length of human Bag-1 3′-UTR was amplified with the forward primer 5′-AAACTCGAGGGTGTAGCAGAAAAAGGCTGTG-3′ and the reverse primer 5′-AAAGCGGCCGCTGTTCTCCAAATATTTATTGAGGTC-3′ from HEK293T cell genomic DNA. The PCR products were cloned into the *Xho*I/*Not*I sites of Psi-CHECK vector (Promega, Madison, WI) and named as Bag-1-UTR-WT. A psi-CHECK-2 construct containing the Bag-1 3′-UTR with mutations in the seed sequence of miR-138 was synthesized using a QuikChange Site-Directed Mutagenesis Kit (Stratagene, Palo Alto, CA) and named as Bag-1-UTR-MUT.

To construct plasmids overexpressing Bag-1, the full coding sequence of Bag-1 was amplified with the primers 5′-CCGGGATCCATGAATCGGAGCCAGGAGG-3′ and 5′-CCGGAATTCTCACTCGGCCAGGGCAAAG-3′ using PCR and then, cloned into the *Bam*HI/*Eco*RI sites of pcDNA3.1 vector (Clontech, Palo Alto, CA). All resulting constructs were verified by sequencing.

### Cell proliferation

Cells were plated at a density of 10^4^ per well in 96-well plates. At different time points (24, 48, and 72 h), 20 μL of 3-(4, 5-dimethylthiazolyl-2)-2,5-diphenyltetrazolium bromide (MTT) (Sigma, St. Louis, MO) was added into each well, and the cells were incubated for another four hours. 200 μL of dimethyl sulfoxide was added to each well after the supernatant was discarded. Optical density was read at the wavelength of 550 nm. Each experiment was repeated at least three times.

### Apoptosis analysis

In order to measure apoptosis, cells were washed with phosphate-buffered saline and trypsinized. Then, they were resuspended in ice-cold 1× binding buffer, and 10 μL of Annexin V-phycoerythrin conjugate (BD Biosciences, Bedford, MA) and 10 μL of propidium iodide solution were added to each group. The apoptotic distribution of the cells in each sample was then determined using fluorescence-activated cell sorting (Beckman Coulter, Fullerton, CA). Results were obtained from at least three independent experiments.

### RNA preparation and quantitative real-time PCR

Total RNA was extracted using TRIzol reagent (Invitrogen) according to the manufacturer’s instructions. To detect the expression of miR-138, stem-loop reverse transcription—polymerase chain reaction (RT-PCR) was performed using a PrimeScript miRNA RT-PCR Kit (Takara, Dalian, China) according to the manufacturer’s instructions. Total RNA (500 ng) was reversely transcribed in the presence of a poly-A polymerase with an oligo-dT adaptor. Quantitative real-time PCR (qRT-PCR) was performed with a forward primer in order to determine the mature miR-138 sequence and a universal adaptor reverse primer. In order to detect the expression of Bag-1, 500 ng of total RNA was reversely transcribed into first-strand of cDNA with M-MLV reverse transcriptase (TakaRa, Dalian, China). The sequences of the primers used were as follows: 5′-TGAGAAGCACGACCTTCATGT-3′ (forward) and 5′-GGAACCCCTATGACCTCTTCA-3′ (reverse). The β-actin sequences of the primers were as follows: 5′-CCCAGATCATGTTTGAGACCT-3′ (forward) and 5′-GAGTCCATCACGATGCCAGT-3′ (reverse). qRT-PCR was performed using an Applied Biosystems 7500 real-time PCR system (Applied Biosystems) and either U6 or β-actin was used as an internal control. Relative quantification of miR-138 and Bag-1 expression was calculated using the 2^-ΔΔCT^ method. All experiments were repeated three times.

### Western blots

Total protein was extracted from cells using 1% RIPA Lysis Buffer (Beyotime, China). Protein concentration was determined using the BCA Assay Kit (Sangon, Shanghai, China). Total protein (50 mg) was separated using 10% sodium dodecyl sulfate-polyacrylamide gel electrophoresis and then, transferred to a polyvinylidene fluoride membrane (Millipore, Billerica, MA). The membrane was blocked with 5% milk for 2 hours at room temperature, followed by an overnight incubation at 4°C with a primary antibodies, such as Bag-1 antibody (Epitomics, Burlingame, CA), Bcl-2, Bax antibodies (CST, Danvers, MA), anti-active caspase-3 antibody (Abcam, Burlingame, CA) and β-actin antibody (Kangcheng, Shanghai, China). The membrane was washed three times in phosphate-buffered saline with Tween-20 and incubated with a goat anti-rabbit horseradish peroxidase secondary antibody for 2 hours at room temperature. Proteins were visualized using the ECL Detection Reagent (Millipore, Billerica, MA), and the signal was detected using an LAS-4000 image analyzer (Fuji Photo Film Co., Tokyo, Japan). The data were normalized to β-actin.

### Luciferase assay and target gene identification

To identify the potential target genes of miR-138, online algorithms TargetScan and PicTar were used. Based on these algorithms, Bag-1 was focused on as a new target of miR-138.

For the luciferase reporter assay, HEK293T cells were seeded onto 24-well plates at 50% confluence before transfection. The cells were then co-transfected with 100 ng of either control vector or miR-138 and 800 ng of either Bag-1-UTR-WT or Bag-1-UTR-MUT using Lipofectamine 2000 (Invitrogen). Reporter assay was performed at 24 hours post-transfection using the Dual Luciferase Assay System (Promega). Transfections were performed in duplicate, and they were repeated at least three times in independent experiments.

### Tumor growth assay in animal models

Twelve four-week-old Balb/c nude mice were purchased from the Shanghai SLAC Laboratory Animal Center (Shanghai, China) and maintained under specific pathogen-free conditions. All experimental procedures involving animals were performed in accordance with the Guide for the Care and Use of Laboratory Animals and were approved by the Animal Experimental Ethics Committee of the Second Military Medical University. NOZ cells control vector (n = 6) and miR-138 (n = 6) were trypsinized, collected by centrifugation, and suspended in RPMI-1640. A 150 μL sample of culture medium containing 1×10^7^ cells was injected subcutaneously into the dorsal flank of each nude mouse. The mice were monitored every week for the growth of tumors, and they were euthanized after 5 weeks. The tumor xenografts were dissected and weighed after the deaths of the mice.

### Statistical analysis

All data are presented as mean ± standard deviation. Two-sample t-tests were performed to compare the expression of miR-138 between gallbladder carcinoma and normal tissues. Statistical analyses were performed using GraphPad Prism 5.0 (GraphPad Software Inc., San Diego, CA). All *P*-values were two-sided, and the significance level was *P*<0.05.

## Results

### MiR-138 represses the expression of Bag-1 by targeting its 3′-UTR

**Fig 1:**
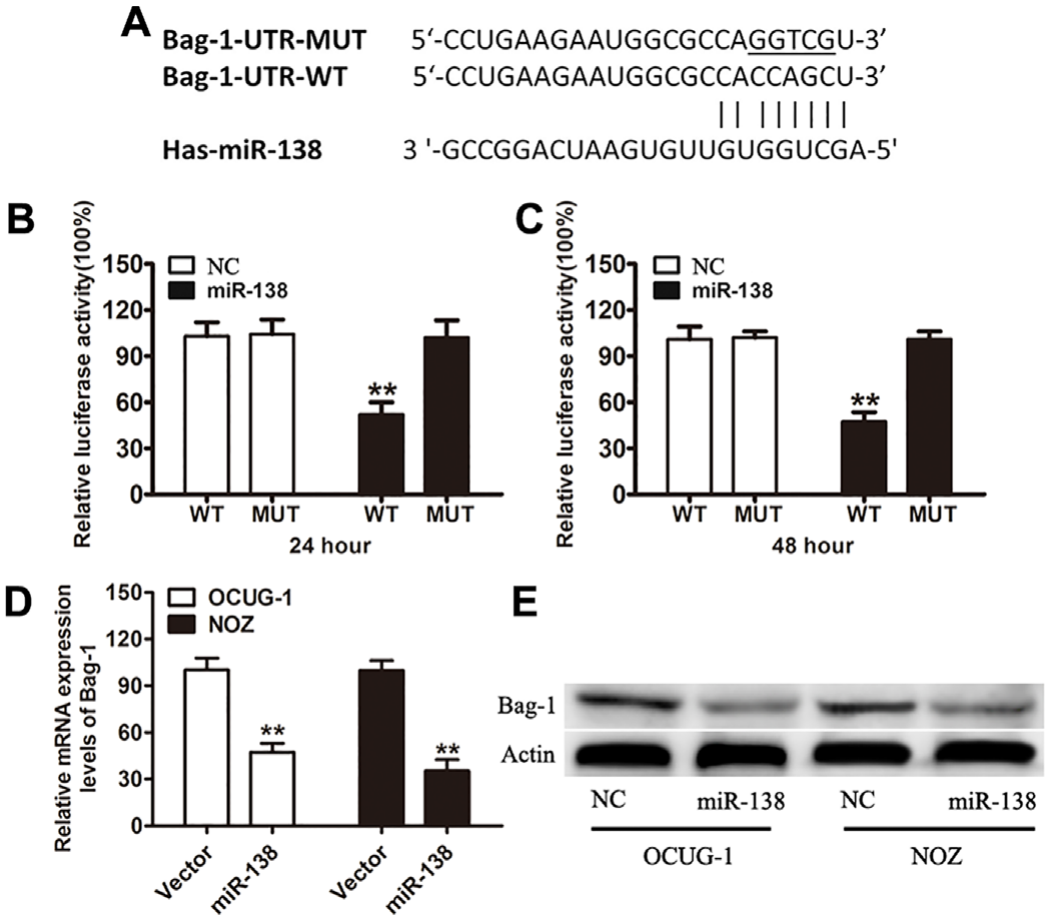
Bag-1 is a direct target of miR-138. (A) A schematic representation showing the putative target site of Bag-1 and mutated target site for miR-138 with the seed region and base substitutions underlined. (B and C) Luciferase reporter assay in HEK293T cells with co-transfection of Bag-1-UTR-WT or Bag-1-UTR-MUT at indicated times. (D and E) The mRNA and protein expression levels of Bag-1 in OCUG-1 and NOZ cells transfected with miR-138 mimic or the control. β-Actin was used as an internal quantitative control. Error bars represent the SD from three independent trials. **P<0.01.

MiR-138 has been reported as one of the known tumor suppressor miRNAs. Bag-1 was identified as one of the candidate effectors of miR-138 based on the putative target sequence of 25–48 bp on Bag-1 3′-UTR (Fig 1A). To examine whether there is a direct interaction between miR-138 and Bag-1 mRNA, the reporter gene assay was performed in 293T cells. It was observed that the relative luciferase activity of the vectors with a miR-138 binding site was significantly decreased in miR-138-transfected cells at 24 and 48 hours. Cells transfected with MUT 3′-UTR were resistant to the suppressor activity of miR-138 (Fig 1B and 1C). These results strongly suggest that miR-138 negatively regulates the expression of Bag-1 by directly targeting its 3′-UTR. Consequently, miR-138 transfected in OCUG-1 and NOZ cells effectively decreased the expression of the endogenous Bag-1 both at mRNA and protein levels (Fig 1D and 1E).

### Expression of miR-138 and Bag-1 in gallbladder carcinoma tissues

**Fig 2:**
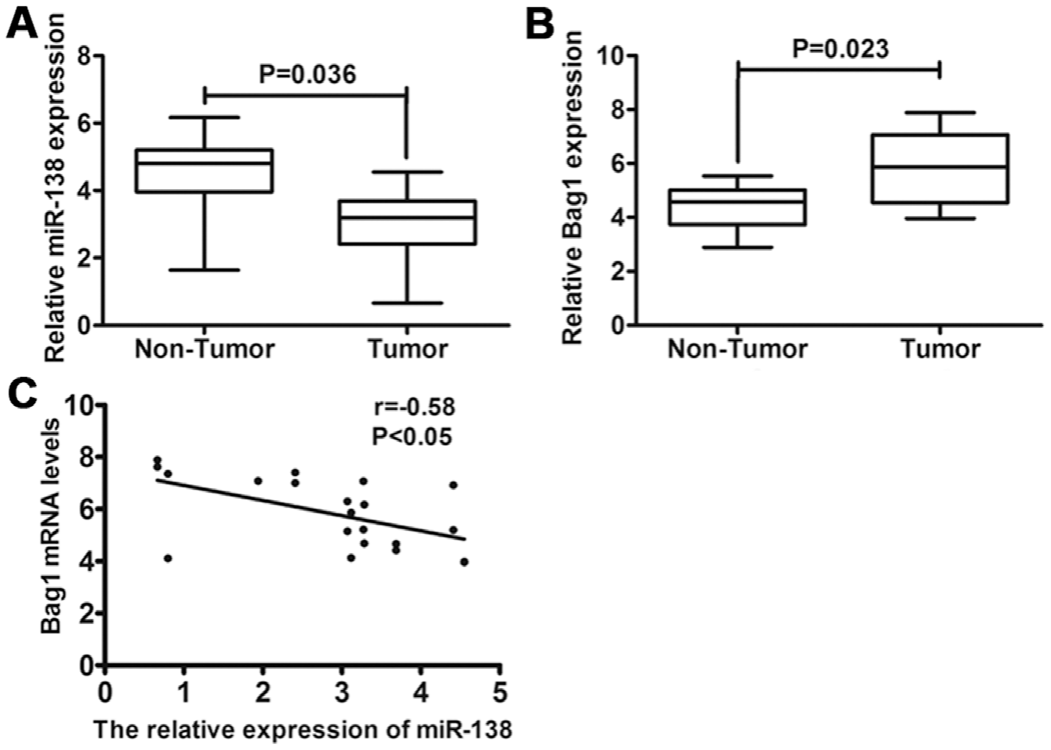
Expression of miR-138 and Bag-1 in gallbladder carcinoma specimens. (A) The expression of miR-138 was determined in gallbladder carcinoma tissues compared with matched normal adjacent gallbladder tissues using qRT-PCR. (B) Relative expression of Bag-1 at mRNA level was examined in gallbladder carcinoma tissues compared with matched normal adjacent gallbladder tissues using qRT-PCR. (C) Inverse correlation between miR-138 and Bag-1 expression in gallbladder carcinoma tissues using Pearson’s correlation coefficient. The expression of miR-138 was normalized to that of U6, and the expression of Bag-1 mRNA was normalized to that of β-actin in each sample.

To investigate the role of miR-138 in gallbladder carcinoma, the expression levels of miR-138 were examined in 49 gallbladder carcinoma specimens and their paired adjacent non-neoplastic tissues. Using qRT-PCR, it was found that the expression levels of miR-138 were reduced in 40 out of 49 (81%) cases of gallbladder carcinoma specimens (*P*<0.001) compared with those of the adjacent non-neoplastic tissues (Fig 2A). Bag-1 has been reported to be upregulated in a number of malignancies including gastric cancer, cervical carcinoma, breast cancer, and colorectal cancer [24–27]. As a result, we assessed the expression of Bag-1 in gallbladder carcinoma and found that Bag-1 was upregulated at the mRNA level in 37 (75%) gallbladder carcinoma tissues compared with adjacent non-neoplastic tissues (*P*<0.001) (Fig 2B). A correlation analysis between the expression of miR-138 and Bag-1 was performed, and the results showed that there is a significant inverse correlation between the mRNA expression levels of miR-138 and Bag-1 (Pearson’s correlation R = −0.58, *P*<0.05) in gallbladder carcinoma (Fig 2C). The data suggests that the reduced expression of miR-138 and increased expression of Bag-1 are frequently observed in human gallbladder carcinoma specimens.

### Overexpression of miR-138 inhibited cell growth and induced apoptosis in gallbladder carcinoma

**Fig 3:**
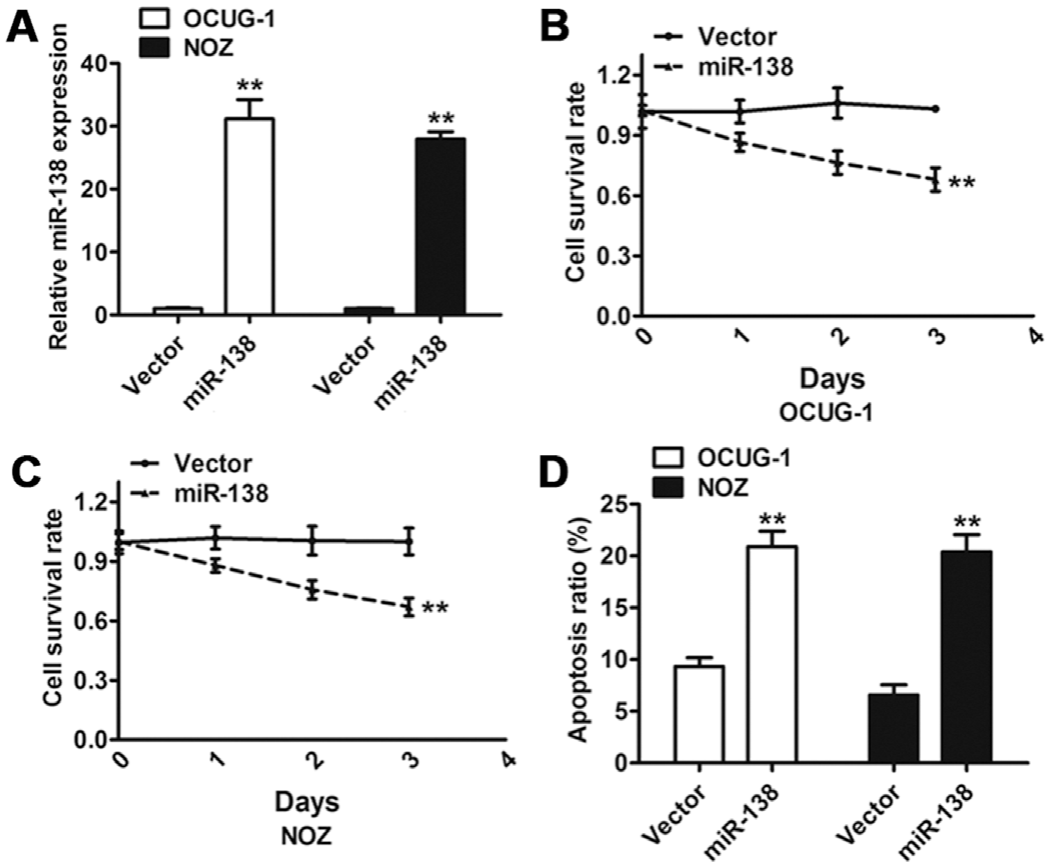
Effect of miR-138 on gallbladder carcinoma cell proliferation and apoptosis. (A) The qRT-PCR analysis confirmed that the expression of miR-138 was clearly increased in cells transduced with miR-138 compared with the control vector. (B and C) Effect of miR-138 on cell proliferation was measured using MTT assay in OCUG-1 and NOZ cells transduced with miR-138 or the control vector. (D) Flow cytometric analysis of the effect of miR-138 on apoptosis of OCUG-1 and NOZ cells. Error bars represented the SD from three independent trials. **P<0.01.

To identify the role of miR-138 in gallbladder carcinoma cell lines, two gallbladder carcinoma cell lines, OCUG-1 and NOZ, expressing low levels of miR-138 were transduced with either the control vector or the miR-138 vector. At 96 hours after transduction, the increased expression level of miR-138 was verified using qRT-PCR in both the OCUG-1 and NOZ cells (Fig 3A). We evaluated the effect of miR-138 on cell proliferation and found a significant reduction in the proliferation rate after overexpression of miR-138 when compared to the control vector (Fig 3B and 3C). This inhibition effect of miR-138 on cancer cell growth was partly due to the increased number of apoptotic cells (Fig 3D). These results indicate that miR-138 regulates cell growth and apoptosis of gallbladder carcinoma cell lines.

### Bag-1 regulated the apoptosis-related genes in gallbladder carcinoma cells

**Fig 4:**
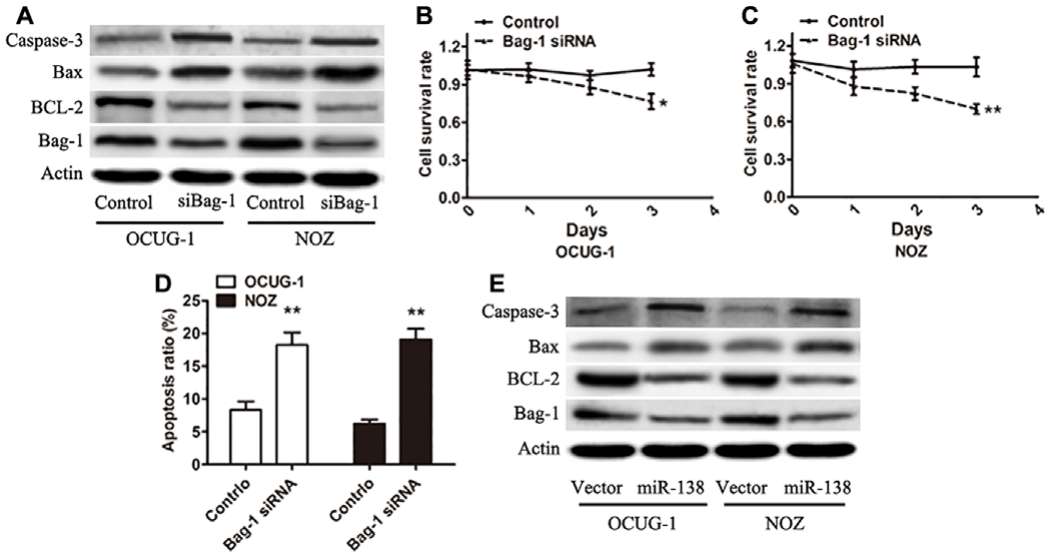
Silencing the expression of Bag-1 could inhibit the proliferation of gallbladder carcinoma cells. (A) Immunoblots were performed to analyze Bag-1, BCL-2, Bax, and cleavage of caspase-3 expression in Bag-1 siRNA-transfected OCUG-1 and NOZ cells. (B and C) MTT assay was used to measure cell proliferative capacity in gallbladder carcinoma cells treated with the control siRNA or Bag-1 siRNA. (D) Gallbladder carcinoma cells transduced with miR-138 compared with the control vector were subjected to Western blot analysis with the indicated antibodies. Error bars represented the SD from three independent trials. *P<0.05; **P<0.01.

To further determine whether Bag-1 is associated with the regulation of apoptosis in gallbladder carcinoma, the expression of Bag-1 protein in OCUG-1 and NOZ cells transfected with the Bag-1 siRNA was analyzed. The results showed a significant decrease compared with that of cells transfected with the negative control siRNA, suggesting that the expression of Bag-1 was effectively inhibited by Bag-1 siRNA. A previous study indicated that Bag-1 is an anti-apoptotic protein that can enhance the anti-apoptotic activity of BCL-2 [28]. The immunoblot data from the present study showed that the expression of BCL-2 was decreased and that the expression of Bax and cleavage of caspase-3 was increased following the silencing of Bag-1 in both these cell lines (Fig 4A). The proliferation capacity of cells transfected with Bag-1 siRNA was significantly inhibited when compared with transfection using the control siRNA (Fig 4B and 4C). Cell apoptosis was analyzed using fluorescence-activated cell sorting. The result found that apoptosis was increased in the Bag-1 siRNA-transfected OCUG-1 and NOZ cells (Fig 4D). We further demonstrated that a decreased expression of BCL-2, increased expression of Bax, and increased cleavage of caspase-3 were found in the miR-138-overexpressing cells, which supports the results attained from silencing Bag-1 (Fig 4E). The results from this study indicate that the target gene of miR-138 Bag-1 can regulate the apoptosis-associated genes in gallbladder carcinoma cells.

### Effects of overexpression of Bag-1 on the proliferation and apoptosis of gallbladder carcinoma cells

**Fig 5:**
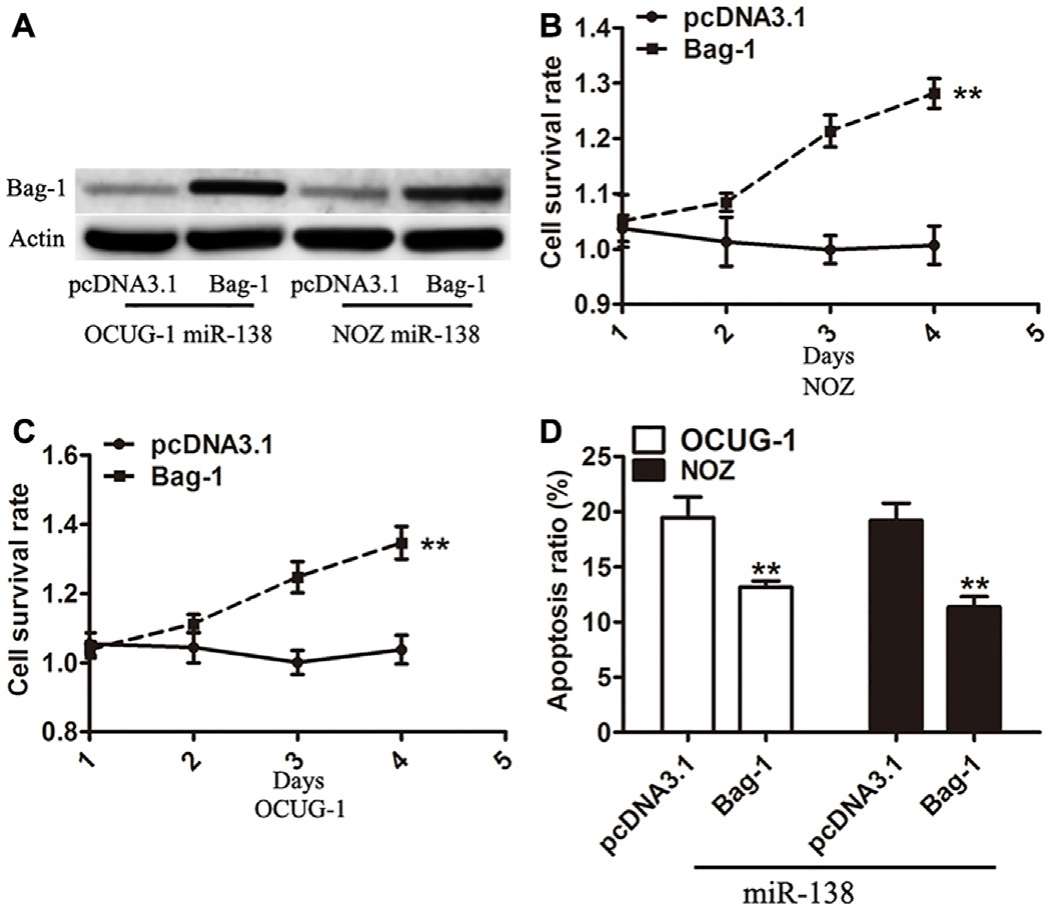
Effects of overexpression of Bag-1 on cell proliferation and apoptosis. (A) Expression of Bag-1 protein in OCUG-1 and NOZ cells stably expressing miR-138 transfected with pcDNA3.1 vector as a control or Bag-1 vector was detected using Western blot at 48 h after transfection. (B and C) Cell proliferation assay was measured in pcDNA3.1 vector or Bag-1-transfected OCUG-1 and NOZ cells stably expressing miR-138. (D) Apoptosis assay was performed after transfection of pcDNA3.1 vector or Bag-1 in gallbladder carcinoma cells. Error bars represented the SD from three independent trials. **P<0.01.

To determine the function of Bag-1, the Bag-1 vector was transfected into the gallbladder carcinoma cells expressing miR-138. Analyses to determine the Bag-1 expression at protein level (Fig 5A) and cell viability were performed (Fig 5B and 5C). The overexpression of Bag-1 significantly reversed the inhibitory effect of miR-138 on the growth of gallbladder carcinoma cells and also, resulted in an inhibition of apoptosis induced by miR-138 (Fig 5D). These data strongly indicate that the biological functions of Bag-1 are correlated to miR-138 in gallbladder carcinoma.

### Overexpression of miR-138 suppressed the growth of tumor *in vivo*

**Fig 6:**
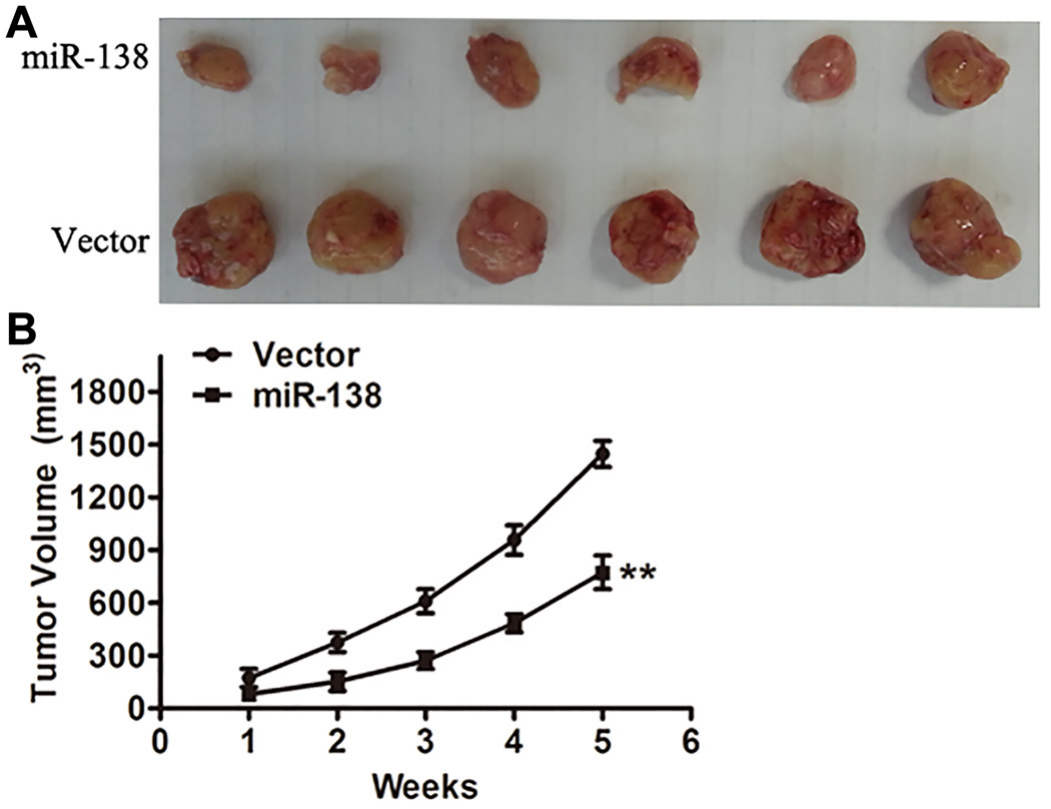
miR-138 inhibits the growth of tumor in vivo. (A) Twelve nude mice were used to establish the gallbladder carcinoma xenograft models. Representative image of tumors formed at the fifth week after injection. Experiments were repeated at least three times. (B) Growth curve was drawn by measuring tumor volumes at the indicated times. **P<0.01.

The effect of miR-138 on cell growth reduction *in vitro* prompted us to further investigate its biological significance *in vivo*. By injecting NOZ cells expressing normal amounts of miR-138 or control vector into the dorsal flank of nude mice, we established the gallbladder carcinoma xenograft models. It was found that the tumors of cancer cells overexpressing miR-138 grew substantially more slowly than the control vector group (Fig 6A). As early as 14 days of post-implantation, the difference in tumor growth between two groups became statistically significant (Fig 6B).

## Discussion

Recent studies indicate that alterations in miR-138 expression are associated with the development and progression of human tumors such as leukemia, nasopharyngeal carcinoma, neuroblastoma, and lung cancer [20–23]. In this study, it was found that miR-138 was frequently decreased in the tumor tissues of patients with gallbladder carcinoma, and restoring miR-138 expression resulted in an inhibition of cell proliferation and an enhancement of cell apoptosis in gallbladder carcinoma. Furthermore, our results showed that miR-138 directly targets Bag-1 by binding its 3′-UTR sites. These results provide the first evidence that miR-138 is downregulated and plays an important role in gallbladder carcinoma.

Some studies have indicated that miR-138 is a master regulator of cell proliferation and apoptosis pathways [20, 23]. Consistent with these results, we found that overexpression of miR-138 significantly inhibits cell proliferation abilities and induces apoptosis *in vitro*. More importantly, NOZ cells transduced with miR-138 showed a significant growth inhibition when the cells were examined in xenograft tumors *in vivo*. The *in vitro* and *in vivo* experiments imply that strategies of introducing miR-138 into cancer cells might have a potential therapeutic value in tumorigenesis.

Numerous studies have shown that Bag-1 is a multifunctional anti-apoptotic protein and that it is involved in several cancer types including gastric cancer, cervical carcinoma, breast cancer, and colorectal cancer [25–29]. However, little is known about the expression and functional role of Bag-1 in gallbladder carcinoma. Here we found a significant elevation of Bag-1 in gallbladder carcinoma tissues compared with the paired non-tumor tissues. Furthermore, a significant inverse correlation was observed between the expression levels of miR-138 and Bag-1 mRNA in the gallbladder carcinoma tissues. Both gain- and loss-of-function studies revealed that Bag-1 is a new target of miR-138.

Because it is not clear whether the negative regulation of Bag-1 by miR-138 contributes to the suppression of gallbladder carcinoma cell proliferation, we silenced Bag-1 with its specific siRNA and found that downregulation of Bag-1 can inhibit cell proliferation and induce apoptosis in gallbladder carcinoma cells. Recent studies indicated that overexpression of Bag-1 is associated with poor prognosis and resistance to doxorubicin in human hepatocellular carcinoma [30]. In neuroblastoma, hTERT was regulated by miR-138 with an increase of Bax expression and decrease of BCL-2 expression [21]. In accordance with these findings, our study confirmed that miR-138 directly targets Bag-1 to regulate the expression of Bax, caspase-3, and BCL-2. Moreover, the significant effects caused by overexpression of miR-138 on gallbladder carcinoma cell growth and apoptosis were reversed partially following restoration of the expression of Bag-1 with a consequential elevation of BCL-2 as well as a suppression of Bax and caspase-3 levels. We concluded that miR-138 directly targets Bag-1 in gallbladder carcinoma cells, thus, suppresses cancer cell growth and induces apoptosis.

Although we partially demonstrated the functions and regulations of miR-138 and its target gene Bag-1, the relationships between the expression level of miR-138 and clinical characteristics and prognosis of patients with gallbladder carcinoma have not yet been investigated. Therefore, more follow-up studies of gallbladder carcinoma patients need to be done.

## Conclusions

The results from the present study showed that miR-138 has a tumor suppressing function through regulation of its target gene Bag-1. These findings will help us understand the role and mechanism of miR-138 in gallbladder carcinoma.

## References

[1] Wistuba II, Gazdar AF. Gallbladder cancer: lessons from a rare tumour. Nat Rev Cancer 2004;4(9):695–706. 15343276 10.1038/nrc1429

[2] Dutta U. Gallbladder cancer: can newer insights improve the outcome? J Gastroenterol Hepatol 2012;27(4):642–653. 10.1111/j.1440-1746.2011.07048.x 22168580

[3] Konstantinidis IT, Deshpande V, Genevay M, Berger D, Fernandez-del CC, Tanabe KK, et al Trends in presentation and survival for gallbladder cancer during a period of more than 4 decades: a single-institution experience. Arch Surg 2009;144(5):441–447; discussion 447. 10.1001/archsurg.2009.46 19451486

[4] Stinton LM, Shaffer EA. Epidemiology of gallbladder disease: cholelithiasis and cancer. Gut Liver 2012;6(2):172–187. 10.5009/gnl.2012.6.2.172 22570746 PMC3343155

[5] Hundal R, Shaffer EA. Gallbladder cancer: epidemiology and outcome. Clin Epidemiol 2014;6:99–109. 10.2147/CLEP.S37357 24634588 PMC3952897

[6] Lahtz C, Pfeifer GP. Epigenetic changes of DNA repair genes in cancer. J Mol Cell Biol 2011;3(1):51–58. 10.1093/jmcb/mjq053 21278452 PMC3030973

[7] Ambros V. The functions of animal microRNAs. Nature 2004;431(7006):350–5. 15372042 10.1038/nature02871

[8] Inui M, Martello G, Piccolo S. MicroRNA control of signal transduction. Nat Rev Mol Cell Biol 2010;11(4):252–263. 10.1038/nrm2868 20216554

[9] Wang H, Zhu Y, Zhao M, Wu C, Zhang P, Tang L, et al miRNA-29c suppresses lung cancer cell adhesion to extracellular matrix and metastasis by targeting integrin beta1 and matrix metalloproteinase2 (MMP2). PLoS One 2013;8(8):e70192 10.1371/journal.pone.0070192 23936390 PMC3735565

[10] Chen CZ. MicroRNAs as oncogenes and tumor suppressors. N Engl J Med 2005;353(17):1768–1771. 16251533 10.1056/NEJMp058190

[11] Calin GA, Croce CM. MicroRNA signatures in human cancers. Nat Rev Cancer 2006;6(11):857–866. 17060945 10.1038/nrc1997

[12] Esquela-Kerscher A, Slack FJ. Oncomirs—microRNAs with a role in cancer. Nat Rev Cancer 2006;6(4):259–269. 16557279 10.1038/nrc1840

[13] Hirata H, Ueno K, Shahryari V, Tanaka Y, Tabatabai ZL, Hinoda Y, et al Oncogenic miRNA-182-5p targets Smad4 and RECK in human bladder cancer. PLoS One 2012;7(11):e51056 10.1371/journal.pone.0051056 23226455 PMC3511415

[14] Huse JT, Brennan C, Hambardzumyan D, Wee B, Pena J, Rouhanifard SH, et al The PTEN-regulating microRNA miR-26a is amplified in high-grade glioma and facilitates gliomagenesis in vivo. Genes Dev 2009;23(11):1327–1337. 10.1101/gad.1777409 19487573 PMC2701585

[15] Ma Y, Yu S, Zhao W, Lu Z, Chen J. miR-27a regulates the growth, colony formation and migration of pancreatic cancer cells by targeting Sprouty2. Cancer Lett 2010;298(2):150–158. 10.1016/j.canlet.2010.06.012 20638779

[16] Liu B, Wu X, Liu B, Wang C, Liu Y, Zhou Q, et al MiR-26a enhances metastasis potential of lung cancer cells via AKT pathway by targeting PTEN. Biochim Biophys Acta 2012;1822(11):1692–1704. 10.1016/j.bbadis.2012.07.019 22885155

[17] Scheibner KA, Teaboldt B, Hauer MC, Chen X, Cherukuri S, Guo Y, et al MiR-27a functions as a tumor suppressor in acute leukemia by regulating 14-3-3theta. PLoS One 2012;7(12):e50895 10.1371/journal.pone.0050895 23236401 PMC3517579

[18] Xu W, Liu M, Peng X, Zhou P, Zhou J, Xu K, et al miR-24-3p and miR-27a-3p promote cell proliferation in glioma cells via cooperative regulation of MXI1. Int J Oncol 2013;42(2):757–766. 10.3892/ijo.2012.1742 23254855

[19] Lezina L, Purmessur N, Antonov AV, Ivanova T, Karpova E, Krishan K, et al miR-16 and miR-26a target checkpoint kinases Wee1 and Chk1 in response to p53 activation by genotoxic stress. Cell Death Dis 2013;4:e953 10.1038/cddis.2013.483 24336073 PMC3877554

[20] Liu X, Lv XB, Wang XP, Sang Y, Xu S, Hu K, et al MiR-138 suppressed nasopharyngeal carcinoma growth and tumorigenesis by targeting the CCND1 oncogene. Cell Cycle 2012;11(13):2495–2506. 10.4161/cc.20898 22739938

[21] Chakrabarti M, Banik NL, Ray SK. miR-138 overexpression is more powerful than hTERT knockdown to potentiate apigenin for apoptosis in neuroblastoma in vitro and in vivo. Exp Cell Res 2013;319(10):1575–1585. 10.1016/j.yexcr.2013.02.025 23562653 PMC3661724

[22] Mitomo S, Maesawa C, Ogasawara S, Iwaya T, Shibazaki M, Yashima-Abo A, et al Downregulation of miR-138 is associated with overexpression of human telomerase reverse transcriptase protein in human anaplastic thyroid carcinoma cell lines. Cancer Sci 2008;99(2):280–286. 10.1111/j.1349-7006.2007.00666.x 18201269 PMC11159409

[23] Zhao X, Yang L, Hu J, Ruan J. miR-138 might reverse multidrug resistance of leukemia cells. Leuk Res 2010;34(8):1078–1082. 10.1016/j.leukres.2009.10.002 19896708

[24] Gao Y, Fan X, Li W, Ping W, Deng Y, Fu X. miR-138-5p reverses gefitinib resistance in non-small cell lung cancer cells via negatively regulating G protein-coupled receptor 124. Biochem Biophys Res Commun 2014;446(1):179–186. 10.1016/j.bbrc.2014.02.073 24582749

[25] Naishiro Y, Adachi M, Okuda H, Yawata A, Mitaka T, Takayama S, et al BAG-1 accelerates cell motility of human gastric cancer cells. Oncogene 1999;18(21):3244–3251. 10359530 10.1038/sj.onc.1202661

[26] Clemo NK, Collard TJ, Southern SL, Edwards KD, Moorghen M, Packham G, et al BAG-1 is up-regulated in colorectal tumour progression and promotes colorectal tumour cell survival through increased NF-kappaB activity. Carcinogenesis 2008;29(4):849–857. 10.1093/carcin/bgn004 18204076

[27] Townsend PA, Cutress RI, Sharp A, Brimmell M, Packham G. BAG-1 prevents stress-induced long-term growth inhibition in breast cancer cells via a chaperone-dependent pathway. Cancer Res 2003;63(14):4150–4157. 12874020

[28] Townsend PA, Cutress RI, Sharp A, Brimmell M, Packham G. BAG-1: a multifunctional regulator of cell growth and survival. Biochim Biophys Acta 2003;1603(2):83–98. 12618309 10.1016/s0304-419x(03)00002-7

[29] Yang X, Hao Y, Ding Z, Pater A, Tang SC. Differential expression of antiapoptotic gene BAG-1 in human breast normal and cancer cell lines and tissues. Clin Cancer Res 1999;5(7):1816–1822. 10430086

[30] Ni W, Chen B, Zhou G, Lu C, Xiao M, Guan C, et al Overexpressed nuclear BAG-1 in human hepatocellular carcinoma is associated with poor prognosis and resistance to doxorubicin. J Cell Biochem 2013;114(9):2120–2130. 10.1002/jcb.24560 23553841

